# Management of Tibial Adamantinoma: Balancing Oncologic Cure and Reconstructive Longevity

**DOI:** 10.7759/cureus.113794

**Published:** 2026-08-01

**Authors:** Yasser Amr, Aye Htun, Simerjit Singh

**Affiliations:** 1 Orthopaedics, Taylor's University, Subang Jaya, MYS; 2 Anaesthesia, Taylor's University, Subang Jaya, MYS

**Keywords:** adamantinoma, bone reconstruction, bone tumors, metastatic bone tumors, recurrence, tibia, vascularized fibular graft

## Abstract

Well-differentiated tibial adamantinoma is a rare, low-grade malignant bone tumor primarily located in the anterior portion of the proximal two-thirds of the tibia and characterized by a biphasic histology. Because of its significant resistance to both radiotherapy and systemic chemotherapy, achieving local control and long-term survival strictly relies on surgical intervention. To evaluate these interventions, a review of English-language literature across PubMed/MEDLINE, Google Scholar, and the Cochrane Library was conducted, focusing on long-term retrospective studies, multicenter series, and case reports. This review assesses oncological and functional outcomes, specifically analyzing the incidence of late tumor recurrence and the durability of complex limb-salvage reconstructive procedures. The literature indicates that en bloc resection with wide safety margins remains the treatment of choice, demonstrating a significantly lower rate of local recurrence compared to intralesional curettage. Although the 10-year overall survival rate is highly favorable, adamantinoma exhibits a characteristically prolonged latency period, with local recurrences and pulmonary or lymphatic metastases commonly emerging 15 to 20 years postoperatively. Furthermore, the extensive intercalary tibial defects resulting from wide resections pose substantial reconstructive challenges. Long-term complications - such as aseptic non-union, deep infection, and progressive structural graft fatigue - are common in limb-salvage constructs utilizing massive structural allografts, vascularized fibular autografts, or hybrid techniques, often necessitating multiple complex revision surgeries. Ultimately, while en bloc resection offers the most consistent oncologic cure despite its reconstructive complexity, effective long-term management mandates lifelong monitoring for delayed metastasis alongside proactive surveillance for mechanical failure in limb-salvage reconstructions.

## Introduction and background

Background

Adamantinoma is a very rare bone tumor with low-grade malignancy and is characterized by a mix of epithelial and osteofibrous stromal components [1,2]. Adamantinoma comprises less than 1% of all primary bone neoplasms and was first recognized in 1900 by Maier. This tumour occurs in multiple distinct subtypes, including dedifferentiated adamantinoma, osteofibrous dysplasia-like (differentiated), and classical adamantinoma [3,4]. Fischer (1913) later reported that these histological features closely resemble those of jaw ameloblastoma [5]. However, a shared histogenic origin between these two entities remains unproven. Some authors have also reclassified this tumour as a malignant angioblastoma [6,7].

Etiology

Adamantinoma has generated substantial debate regarding its histogenic origins, with theories proposing synovial, endothelial, or epithelial derivation [1,6,8]. While earlier theories posited congenital epithelial displacement or traumatic implantation [2,3,5], others suggested an origin from primitive vascular mesenchymal cells [1,6,9]. Today, controversy remains regarding the potential developmental sequence linking benign osteofibrous dysplasia to classical adamantinoma [4,5].

Presentation

Adamantinoma typically occurs between the second and fifth decades of life and shows a slight male predominance (male: female ratio 5:4). Approximately 97% of cases involve long bones, most commonly the tibia in 80-85%, with concurrent fibular involvement in 10-15% of cases [3,4]. Less frequently, it affects other bones, including the ulna, radius, ribs, humerus, femur, spine, pelvis, and the small bones of the hands and feet [1,5,10]. Aside from the appendicular skeleton, adamantinoma may rarely develop in axial regions, including two occurrences documented in the ribs [11,12]. Dini et al. summarized a case of spinal adamantinoma and analyzed five earlier reports, two of which occurred simultaneously in the mandible [13]. Additionally, Mosher reported a maxillary sinus case and instances in the pituitary gland and in the mandible [14]. Furthermore, multiple reports have identified soft tissue adamantinomas arising without any skeletal involvement. Bambirra et al., Bertoni et al., Mills and Rosai, and Keeney et al. described five pretibial soft tissue adamantinoma cases with histological features resembling those of the osseous form and proposed a potential link between them [10,15-17].

Clinical features

The initial manifestation of adamantinoma is insidious and non-specific. As the disease progresses slowly, patients frequently experience symptoms for several years before seeking medical advice. About 60% of the patients reviewed by Moon and Mori (200 cases) were said to have a history of significant trauma [2]. Patients generally present with localized swelling and tenderness, with or without pain, as well as impaired weight-bearing, physical deformity, and erythema [18-20]. Paraneoplastic hypercalcemia and pancreatitis have been documented in some case reports involving tibial adamantinoma with pulmonary metastatic disease [4,5]. In cases of spinal involvement, patients may present with neurologic deficits in addition to pain [21].

Differential diagnoses

When evaluating a suspected adamantinoma of the long bones, it is important to rule out several benign and malignant conditions. Differentiating adamantinoma from these mimics relies heavily on patient age, anatomic location within the tibia, characteristic cortical "soap-bubble" lucencies on imaging, and the definitive biphasic epithelial-stromal profile on histology. A comprehensive comparison of these overlapping features is summarized in Table 1.

**Table 1:** Differential diagnosis of tibial adamantinoma: clinical, radiographic, and histopathological distinguishing features. Summary of the principal differential diagnoses of tibial adamantinoma, highlighting overlapping clinical and radiographic features together with key distinguishing characteristics based on imaging, histopathology, and immunohistochemistry. The differential diagnoses include osteofibrous dysplasia, fibrous dysplasia, chondromyxoid fibroma, aneurysmal bone cyst, osteomyelitis, eosinophilic granuloma, Ewing's sarcoma, osteosarcoma, epithelioid hemangioendothelioma, hemangiosarcoma, and metastatic carcinoma. Accurate diagnosis requires correlation of clinical presentation, imaging findings, histopathological examination, and immunohistochemical profiling.

Differential Diagnosis	Clinical/Radiographic Overlap	Key Distinguishing Features
Osteofibrous Dysplasia (OFD)	Typically occurs in the anterior tibial cortex; presents with radiolucent, multilocular lesions.	Benign, often regresses spontaneously after skeletal maturity; histologically lacks the large, cohesive, clustered epithelial islands of classic adamantinoma. Immunohistochemistry shows only scattered, isolated cytokeratin-positive cells without structural nesting.
Fibrous Dysplasia	Expansile, radiolucent lesion within the bone; can cause bowing or deformity of the tibia.	Ground-glass appearance on radiography; histologically shows irregular "Chinese letter" woven bone without an epithelial component.
Chondromyxoid Fibroma	Eccentric, radiolucent lesions in the metaphysis of long bones with sclerotic margins.	Lacks biphasic osteofibrous/epithelial histology; contains lobules of chondroid, myxoid, and fibrous elements.
Aneurysmal Bone Cyst (ABC)	Expansile, multiloculated "soap bubble" appearance on imaging; causes localized swelling.	Fluid-fluid levels typically visible on MRI; lacks the dense fibrous stroma and epithelial cells; often secondary to other lesions.
Osteomyelitis	Lytic lesion with cortical destruction, periosteal reaction, and localized pain/swelling.	Clinical signs of infection (e.g., fever, elevated inflammatory markers); MRI shows extensive bone marrow edema and potential abscess formation.
Eosinophilic Granuloma	Localized pain; lytic bone lesions with potential periosteal reaction and endosteal scalloping.	Sharp, "punched out" lesions on imaging; mostly in younger patients; histologically features Langerhans cells positive for CD1a and Langerin.
Ewing's Sarcoma	Diaphyseal location in long bones; causes localized pain, swelling, and cortical destruction.	Highly aggressive with rapid progression; exhibits an "onion skin" periosteal reaction; characterized by small round blue cells.
Osteosarcoma	Malignant bone tumor usually in the long bones; causes pain, swelling, and cortical destruction.	Highly aggressive; exhibits a "sunburst" periosteal reaction or Codman's triangle; histologically defined by the production of malignant osteoid.
Epithelioid Hemangioendothelioma	Rare vascular tumor that can present as an expansile, lytic lesion in long bones.	Endothelial origin confirmed by vascular markers (CD31, CD34, ERG) and CAMTA1 fusion; completely lacks the cytokeratin-positive epithelial islands characteristic of adamantinoma.
Hemangiosarcoma	Destructive, aggressive lytic lesion; presents with pain and rapidly enlarging mass.	Highly malignant vascular tumor; marked cytologic atypia with anastomosing vascular channels; strongly positive for CD31 and factor VIII.
Metastatic Carcinoma/Epithelial Metastases	Destructive, osteolytic lesions; can test positive for epithelial markers (cytokeratins), mimicking adamantinoma.	Older adult demographic; history of primary visceral malignancy. While strongly cytokeratin-positive, it lacks the characteristic osteofibrous stroma (zoning architecture) of adamantinoma and exhibits high-grade cytological atypia/mitotic activity.

Radiological findings

**Figure 1:**
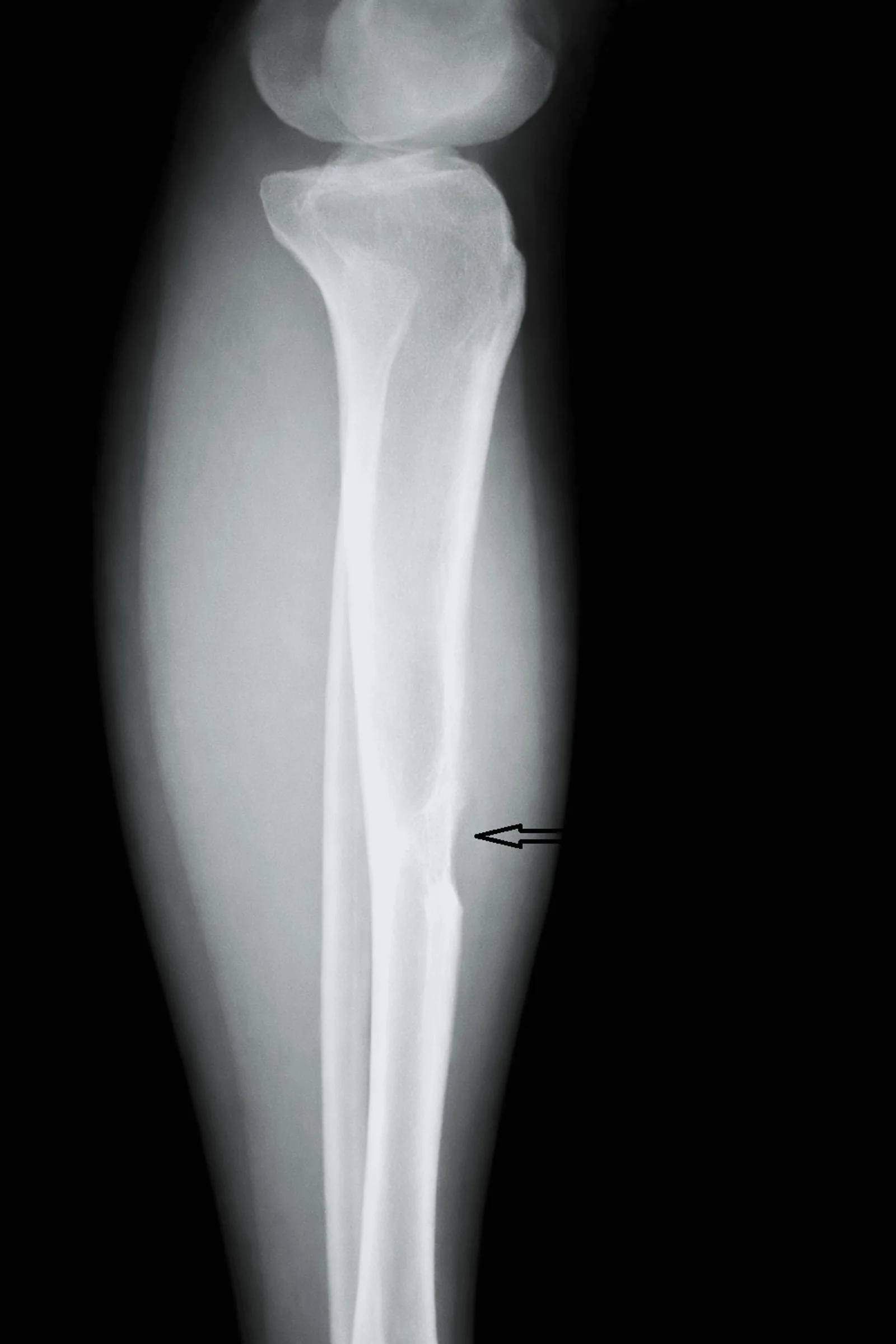
Lateral radiograph of the left tibia demonstrating adamantinoma. The radiograph displays the lateral aspect of the adult tibia and fibula. The black arrow points to an area of cortical irregularity and localized radiolucency on the anterior surface of the tibial shaft, consistent with adamantinoma. A soft tissue shadow of the leg is visible. Note the distinct appearance of the anterior cortical involvement and subtle bone changes within the diaphysis. Source: Original image owned by the authors.

**Figure 2:**
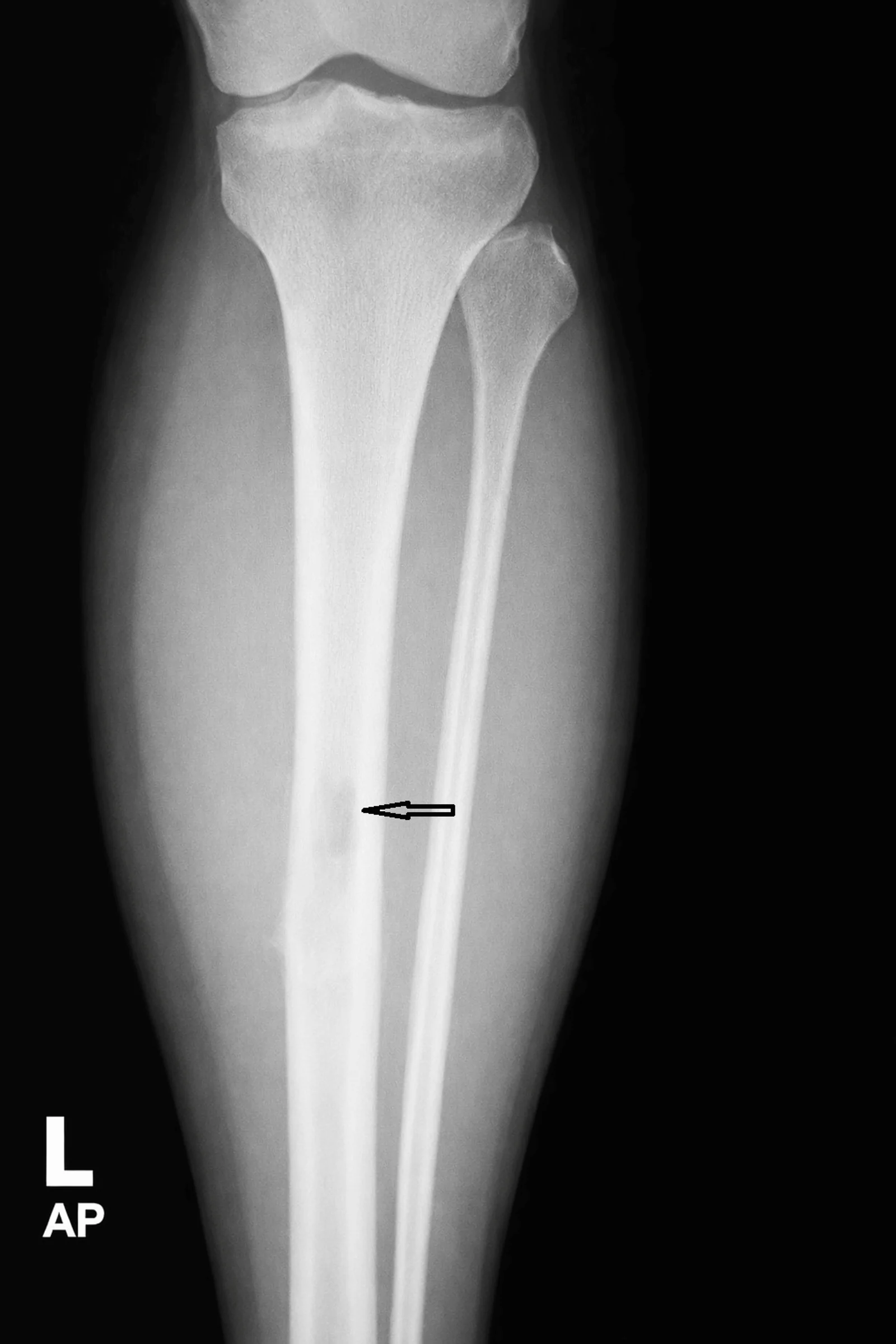
Anteroposterior (AP) radiograph of the left tibia demonstrating adamantinoma. The radiograph displays the AP aspect of the adult left tibia and fibula. The black arrow points to a well-defined, eccentric, expansile lytic lesion located in the diaphysis of the tibial shaft. The lesion has a slightly ground-glass appearance and is surrounded by a thin, sclerotic rim, consistent with imaging characteristics of an adamantinoma. Soft tissue structures and the adjacent fibula are visible. Note the specific medullary location. Source: Original image owned by the authors.

Jain et al. observed that a lesion including the diaphysis, involving the anterior cortex and extending into bone marrow, is indicative of adamantinoma. Radiographic evaluation commonly reveals characteristic features of the tumour, with concurrent pathological bone fractures occurring in approximately 16-23% of cases [3,18]. Typically, the tumor appears as a single, multilocular osteolytic lesion positioned either centrally or eccentrically, with sharply demarcated, sclerotic borders indicative of a slow-growing process (Figures 1-2).

Advanced lesions demonstrate endosteal scalloping, cortical thinning, and eventual cortical breach. The presence of overlapping radiolucencies may create a soap-bubble pattern within the mid or distal portions of the diaphysis and metaphysis [5]. The mass may be expansile, with ill-defined margins and internal septations; however, homogeneous enhancement and infiltration of the medullary canal are usually limited to the intra-cortical areas. In about 15% of cases, the lesion may invade the surrounding soft tissues. A concomitant fracture may cause periosteal reaction, and the lesions may occur as nodular foci [1,5]. The diagnosis can also be refined with other radiological modalities. CT offers contributions in the assessment of cortical bone involvement and for lesions with osseous metastases, but MRI is considered the gold standard. MRI has better contrast resolution, which provides clearer separation of foci that are more distant, as well as soft tissue involvement and the effect on the margin of intramedullary tumor. MRI is also useful for staging and planning surgical interventions. The utilization of imaging agents, particularly in the case of nuclear medicine where increased vascularity and pooling around the lesion are often seen, with technetium-99m methylene diphosphonate (MDP) accumulation. Positron emission tomography (PET) detects fluorodeoxyglucose (FDG)-avid lesions at additional sites in the body, which facilitates thorough assessment of the overall disease burden [3,5,22].

Histopathological diagnosis

Histopathological diagnosis by biopsy should be comprehensive and aimed specifically at the radiolucent areas within the lesion. Image-guided techniques, such as computed tomography (CT) or ultrasound, may further improve the process by allowing for accurate sampling of tissues. Histopathological findings should be interpreted in conjunction with radiological data, keeping in mind that the intrinsic heterogeneity of tumours may limit diagnostic accuracy. This highlights the complexities of this disease and emphasizes that specific knowledge for diagnosis is needed with a thorough clinical evaluation. Therefore, the multidisciplinary team (MDT) is essential to guide and improve patient management [3,4]. Due to its biphasic architecture, adamantinoma features epithelial cell nests contained within osteofibrous stroma. The epithelial component typically demonstrates trabecular, nested, or cord-like architectural patterns, with cytologic features ranging from basaloid differentiation to squamous maturation [1-3]. Immunohistochemistry reveals robust positivity for cytokeratins (CK5, CK14, and CK19) and epithelial membrane antigen (EMA), establishing an epithelial origin [3,23]. Two major histological patterns are recognized: (1) Classic adamantinoma: characterized by well-circumscribed islands of epithelium within a dense fibrous stroma, which occurs primarily in adult patients; and (2) osteofibrous dysplasia-like adamantinoma: often seen in the younger population, contains smaller epithelial nests throughout a fibro-osseous matrix that often mimics osteofibrous dysplasia. The stromal cells exhibit positivity for vimentin. Immunohistochemical studies, including flow cytometric analysis and assessment of p53 expression, have demonstrated p53 protein positivity in up to 48% of classical adamantinoma subtypes [24]. The lesion is typically well-circumscribed but exhibits infiltrative growth at the cortical margins. Few mitotic figures are seen, indicative of its generally indolent behaviour; however, even in instances of recurrence or incomplete surgical resection, progression to more aggressive phenotypes could be observed. Because osteofibrous dysplasia is a less aggressive benign lesion that may regress spontaneously, histopathological differentiation is very important [25].

## Review

Methodology

Search Strategy and Databases

A comprehensive literature search was conducted to identify relevant clinical studies, case series, and reports regarding the surgical management and oncological outcomes of tibial adamantinoma. The primary academic databases queried included PubMed/MEDLINE, Google Scholar, and the Cochrane Library. The search encompassed literature published from database inception up to 2026, ensuring the capture of both foundational historical reports and recent international multicenter analyses.

Keywords and Search Terms

The literature search utilized various combinations of Medical Subject Headings (MeSH) and free-text terms, including "Adamantinoma," "Tibial Adamantinoma," "malignant bone tumor," "limb salvage," "en bloc resection," "intercalary reconstruction," "distraction osteogenesis," "vascularized fibular graft," "tumor recurrence," and "metastatic adamantinoma." Boolean operators (AND, OR) were employed to refine the search queries and focus specifically on the intersection of oncological control and reconstructive longevity.

Inclusion and Exclusion Criteria

Inclusion criteria included full-text articles published in the English language; retrospective cohort studies, multicenter case series, clinical reviews, and well-documented case reports; studies explicitly detailing surgical management (e.g., limb salvage, en bloc resection, amputation), reconstructive modalities, hardware survivorship, local recurrence rates, or long-term follow-up protocols for tibial adamantinoma.

Exclusion criteria included articles published in languages other than English without available full-text translations; in vitro, biomechanical, or animal studies; conference abstracts or posters lacking full-text manuscript data; studies with insufficient clinical follow-up or those lacking clear histopathological differentiation between classic adamantinoma and isolated osteofibrous dysplasia.

Data Extraction and Synthesis

Initial screening was performed by reviewing the titles and abstracts of the identified records to determine relevance. Selected full-text articles were subsequently evaluated to extract critical clinical data, including patient demographics, tumor location, surgical margin status, types of reconstructive constructs, structural complications (e.g., aseptic non-union, graft fracture), and long-term oncological outcomes (time to local recurrence and distant metastasis). Given the extreme rarity of adamantinoma and the heterogeneity of the available data, a narrative synthesis approach was adopted to systematically summarize treatment paradigms, reconstructive challenges, and surveillance strategies.

Metastatic disease

Lymphovascular channels are responsible for metastases in around 15-30% of patients. Lungs and regional lymph nodes are the most common sites of metastasis. Bone and abdominal viscera involvement are less frequently noted, while retroperitoneal dissemination is rarely seen [18,26]. A significant difference was noted in the reported incidence of lymphatic metastases. Keeney et al. recorded a 7% risk while Moon and Mori found significantly higher rates of up to 28.6% in their autopsies [2,10]. Recurrences and metastases might occur over extended durations, with instances documented to happen up to 36 years after the initial therapy. Schowinsky et al. reported a patient who developed a possible secondary brain metastasis originating from a metastatic lung lesion 32 years after surgical resection of tibial adamantinoma [22,27]. Giannoulis et al. reported a case with a 46-year-old patient who had several early recurrences in the tibia, followed by late metastases to the lung and ribs that manifested 13 years post-diagnosis. However, the long pattern of recurrence and relatively low-grade malignant potential of adamantinoma are not all-encompassing. Binesh et al. (2012) discussed a case of a 19-year-old patient who died within weeks of diagnosis and initial surgical resection of an adamantinoma in the pelvis resulting from severe liver failure due to extensive metastases [4,19,28]. Another critical point is to be alert for incidental, unrelated primary synchronous malignancies. To date, there have only been two such cases reported in the literature: one case describes metastatic chromophobe-type renal cell carcinoma discovered after a partial nephrectomy initially done for presumed adamantinomatous metastasis, and another describes simultaneous metastatic adamantinoma with a primary breast cancer [26]. This aspect further highlights the significance of the multidisciplinary team in the process of decision-making for diagnosis as well as treatment of such lesions. When evaluating suspected metastases, major challenges arise in interpreting investigations related to the disease's location and origin. Clinicians should also consider excluding genetic susceptibility to malignancy in these patients [3,22].

Local recurrence

Local recurrence rates at five and 10 years by Kaplan-Meier analysis were 8.6% and 18.6%, respectively. There was no statistically significant association between tumor stage and this recurrence rate [4,8,29]. Of importance, recurrences have been presented 36 years from resection, necessitating radical excision and accurate long-term follow-up. Margin status is the most consistent predictor of recurrence, and wide en bloc resection demonstrates the best long-term disease control among prognostic factors.

Surgical management and reconstructive modalities

Patients are generally treated under the care of a sarcoma MDT, with primary management provided by orthopedic oncologists and pulmonary lesions managed by thoracic surgeons. Evidence synthesis consistently demonstrates that extensive en bloc resection with wide, negative margins is the established gold standard for tibial adamantinoma. Intralesional treatments or curettage are contraindicated, as they are linked to local recurrence rates exceeding 80-90% [2,10,28,30]. Because the level of excision depends on tumor dimensions, cortical involvement, and soft-tissue expansion, achieving negative margins often leads to substantial segmental defects that require complex restoration.

Reconstruction following extensive tibial resection has evolved considerably. Current evidence highlights four primary modalities, each with distinct functional outcomes and long-term trade-offs:

Biological Reconstruction

In pediatric and younger adult populations, massive structural allografts or vascularized fibular autografts provide excellent long-term functional outcomes once biological incorporation is achieved. These grafts offer superior potential for biological remodeling and mechanical resistance to fracture over a patient's lifespan. However, clinical evidence highlights that the consolidation process is slow and fraught with early challenges; structural complications such as aseptic non-union and graft fracture remain significant concerns, occurring in up to 23% of massive graft cases [31,32].

Mechanical Reconstruction

Endoprosthetic replacements afford immediate structural stability, allowing for early post-operative weight-bearing and faster initial rehabilitation. While short-term functional outcomes are favorable, long-term survivorship data indicate high failure rates over time. Complications such as progressive prosthetic loosening, mechanical fatigue, and deep infection frequently mandate complex revision surgeries, making this modality less ideal for younger patients with long life expectancies [33,34].

Distraction Osteogenesis (Bone Transport)

This method bridges a large segmental bone defect by gradually advancing healthy bone segments across the gap. The primary outcome advantage is the enhancement of autologous regenerate bone formation, offering a highly durable biological result once completed. However, clinical limitations include a prolonged time in external fixation and a high risk of non-union at the docking site, which frequently requires supplementary internal fixation and bone grafting to resolve (Table 2) [35].

**Table 2:** Comparison of reconstructive strategies following wide resection of tibial adamantinoma. Comparison of the principal reconstructive options after wide excision of tibial adamantinoma, including vascularized fibular grafts, endoprosthetic replacement, distraction osteogenesis (bone transport), and hybrid dual reconstruction techniques. The table summarizes the key advantages, major limitations, and potential complications, and the clinical scenarios in which each reconstructive modality is most appropriately applied. Selection of the optimal reconstructive method should be individualized based on patient age, defect size, functional requirements, comorbidities, and anticipated long-term outcomes.

Modality	Key Advantages	Primary Limitations and Complications	Ideal Patient Profile
Biological (Vascularized Fibular Grafts)	Excellent biological incorporation; high potential for remodeling and mechanical resistance to fracture.	Slow consolidation process; risk of graft failure and aseptic non-union.	Pediatric and younger adult populations with longer life expectancies.
Mechanical (Endoprosthetic Replacement)	Immediate structural stability; allows for early post-operative weight-bearing.	Poor long-term implant survival; high risk of prosthetic loosening, fatigue, and deep infection.	Older patients or those requiring immediate mobility.
Distraction Osteogenesis (Bone Transport)	Enhances autologous regenerate bone formation; bridges large segmental gaps biologically.	Prolonged external fixation time; risk of non-union at the docking site requiring further grafting.	Patients with massive segmental defects suitable for gradual transport.
Hybrid Dual Approaches	Attempts to strike an optimal balance between immediate mechanical stability and long-term biological durability.	Technically demanding; carries compounding risks of both biological and mechanical failures.	Patients requiring both early stability and durable biological integration.

Hybrid Approaches

Hybrid constructs - such as combining a massive structural allograft with a vascularized fibular autograft, or using biologic grafts alongside intercalary endoprostheses - have emerged in an attempt to optimize clinical outcomes. Evidence suggests these dual approaches successfully bridge the reconstructive gap, providing the immediate mechanical stability of an implant alongside the long-term biological integration of an autograft, though they remain technically demanding [34].

Complications and survivorship

Although limb salvage continues to be the mainstay of management in most patients, reoperation rates may be high, with one review reporting 39%. This high frequency is largely due to the occurrence of surgical complications [8]. Qureshi et al. conducted a 10-year retrospective case study with 70 patients from 23 cancer institutions. A total of 91% underwent limb salvage, while 51% received intercalary allograft reconstructions; ultimately, an 84% preservation rate was maintained, given that 8% of cases subsequently necessitated amputation. Additionally, wide surgical margins were successfully secured in 92% of the procedures. About 48% of cases developed complications related to reconstruction, most frequently allograft fracture (24%) and non-union (23%). Other rare complications included infection (10%), unidentified soft tissue problems (3%), and delayed union (3%). Adamantinoma is known for its local aggressiveness. Kaplan-Meier analysis demonstrated local recurrence rates of 8.6% at five years and 18.6% at 10 years, with no statistically significant association between recurrence and disease stage [4,8,29]. In a study by Houdek et al., 10-year disease-specific survival reached 92%, with a recurrence-free survival rate of 72% [8].

To optimize these outcomes, the literature clearly indicates that primary amputation does not confer a survival advantage over wide resection. Consequently, amputation is generally reserved for instances of local recurrence, situations where limb salvage is anatomically unfeasible, or in the event of severe, intractable reconstructive complications [28].

Finally, the European Musculoskeletal Oncology Society (EMSOS) recently published an international multicenter investigation of 190 adamantinoma cases spanning three decades - the largest case series on record. Evaluating long-term survival and surgical outcomes, the authors reported a 24% overall local recurrence rate, driven by factors including occult skip lesions, periosteal involvement, and pathological fractures. These findings strongly synthesize the core reconstructive dilemma: the absolute necessity of aggressive, wide surgical resection with negative margins to ensure oncologic survival, directly resulting in massive defects that demand highly durable reconstructive solutions [18].

Adjuvant therapy

Given the strong resistance of adamantinoma to traditional chemotherapy and radiotherapy, systemic treatment options have historically been limited. Although subsequent literature has expanded upon early foundational studies, the inherent chemoresistance of adamantinoma means that the primary role of medical and clinical oncologists remains centered on managing advanced or metastatic disease. The authors stress that this conclusion is based on a summary of case series and observational reports identified in their literature review and not data derived from clinical trials, which are limited because of the rarity of this pathology [9,18]. Hazelbag et al. described three patients with metastatic disease who received chemotherapy without any measurable reduction in tumour burden or improvement in survival. Similarly, three other authors reported only temporary disease stabilization following chemotherapy and radiotherapy, with progression occurring within a few months in three additional cases [36-39]. Although systemic chemotherapy regimens like etoposide and cisplatin have been used for pulmonary metastases, these therapies often have modest activity coupled with significant toxicity limiting their clinical use. Highlighting a rare therapeutic success, Lokich has documented an evident case of disease regression. In this instance, a patient with pulmonary metastases from a primary bone adamantinoma demonstrated clinical response on two distinct occasions to etoposide-based chemotherapy, paired initially with cisplatin, and subsequently with carboplatin, following an additional positive response to palliative radiotherapy; the patient achieved a prolonged metastatic disease course exceeding three years [40]. Recently, the therapeutic strategy for pulmonary metastatic adamantinoma has shifted toward targeted options, particularly tyrosine kinase inhibitors (TKIs), as supported by a growing body of case literature. Specifically, multi-targeted agents like sunitinib and pazopanib have successfully induced partial responses or achieved disease stabilization in patients with advanced, unresectable disease. Broader clinical reviews reinforce that although the overall prognosis for metastatic adamantinoma remains poor, these targeted systemic therapies currently represent the most promising frontier for managing inoperable cases [36,38,41].

Prognosis

Poorer clinical outcomes have been associated with other factors, including male gender; a short duration of painful symptoms prior to diagnosis; younger age at diagnosis; absence of squamous differentiation on histology; and intraregional treatment modes [10,27]. Additionally, the EMSOS concluded that local recurrence risk was greatly impacted by resection edges, patient gender, and pathological fracture [18]. Local recurrence risk is not significantly affected by tumor stage, biopsy type, or grafting repair process. In Aytekin et al.'s study of 92 patients, survival periods were analyzed, along with patient race, year of diagnosis, and primary tumor site. Another case of adamantinoma presenting with severe hepatic and pulmonary metastases was retrospectively evaluated; the authors noted an aggressive histological variant marked by prominent large-cell growth, a lack of classic stroma, and complete epithelial predominance [4,29,30,42]. This highly aggressive behaviour underscores that while overall mortality from adamantinoma is reported in up to 11% of cases, histological architecture dictates individual risk [18]. In a recent analysis of 92 patients utilizing Surveillance, Epidemiology, and End Results (SEER) data from US public cancer registry records spanning 1975 to 2016, with a mean follow-up of 138 ± 90.3 months, data demonstrated a five-year overall survival rate of 98.8%. The 10-year overall survival rate for this cohort, while still undergoing long-term evaluation, is estimated at 91.5%. Based on these results, it was concluded that adamantinoma patients have a good prognosis [42].

Follow-up

Due to its high recurrence rate and tumor metastases, all patients require long-term follow-up, with some experts recommending lifelong follow-up, yet there remains a significant lack of data on the ideal imaging study or follow-up interval [3,10,19].

Houdek et al. noted that 42% of patients with distant metastatic disease have isolated metastases to either abdominal recurrences or pelvic lymphadenopathy, supporting the role for abdominopelvic CT or PET imaging in these patients. Recently, the authors have put forth a diagnostic algorithm based solely on MRI of the involved limb coupled with chest CT over 10 years [8]. The majority of the authors recommend serial clinical/radiographic follow-up, most stressing MRI or radiographs of the involved limb. Some also recommend surveillance chest X-ray, CT chest, or whole-body MRI. The EMSOS especially highlights this with a recommended follow-up for 20 years or longer. This is supported by their finding that 11% of local recurrence cases were diagnosed beyond 10 years since the initial treatment and demonstrates the benefit of continued imaging in both the chest and surgical site up to a decade or more following intervention by either radiographs or MRI. Another aspect is to emphasize the need for a tailored action plan, varying in extent, based on each patient's disease progression so far (Table 3) [4,20,22,27].

**Table 3:** Recommended long-term surveillance protocol following surgical treatment of tibial adamantinoma. Proposed long-term surveillance protocol following surgical treatment of tibial adamantinoma. Proposed postoperative surveillance schedule for patients treated with limb-salvage surgery for tibial adamantinoma. This original framework was synthesized by the authors based on current clinical recommendations and literature [4,20,22,27]. The protocol outlines the recommended frequency of follow-up, imaging modalities, and clinical objectives.

Surveillance Phase	Frequency	Primary Modality and Site	Clinical/Structural Rationale
Early Post‑operative (Years 1-2)	Every three months	• Plain Radiographs (Tibia/Fibula) • Chest CT • Physical Examination	• Monitor early graft incorporation and bone healing at docking sites. • Screen for rapid‑onset mechanical hardware failure or early structural complications. • Catch aggressive baseline micrometastases.
Intermediate Follow‑Up (Years 3-5)	Every six months	• Plain Radiographs (Affected Limb) • Advanced MRI (Affected Limb) • Chest CT	• Detect early‑to‑mid-term local soft‑tissue or osseous recurrences. • MRI provides high‑contrast resolution to check for subtle cortical margins and intramedullary changes. • Evaluate long‑term graft remodeling/hypertrophy progress.
Late Follow‑Up (Years 6-10)	Annually	• Plain Radiographs (Affected Limb) • Chest CT (or Surveillance Chest X‑ray)	• Screen for delayed pulmonary and regional lymphatic metastases, which are characteristically common in this indolent window. • Assess for progressive structural graft fatigue or aseptic hardware loosening.
Extended Surveillance (Years 11-20+/Lifelong)	Every one to two years	• Plain Radiographs (Affected Limb) • Chest CT or Abdominopelvic CT/PET (if symptomatic)	• Essential due to documented late recurrences stretching up to 36+ years. • Monitor long‑term survivorship of the limb‑salvage construct. • Screen for potential late‑onset visceral or pelvic lymphadenopathy dissemination.

Limitations of the study

This review is subject to several limitations, largely attributable to the rarity of adamantinoma and the consequent scarcity of high-quality evidence. Most available studies consist of small retrospective case series and observational reports, limiting the ability to formulate definitive evidence-based management guidelines. Crucially, the inherent clinical heterogeneity of these retrospective reports, combined with highly fragmented sample sizes and a lack of standardized quantitative reporting across decades, precluded the use of formal statistical summary methods such as meta-analysis or meta-regression. Consequently, pooled quantitative outcomes, confidence intervals, and effect sizes could not be reliably calculated, limiting the statistical rigor of the synthesis. Furthermore, considerable variability in follow-up duration and surveillance protocols among published studies hinders the establishment of standardized recommendations regarding the optimal frequency and imaging modalities for long-term monitoring. In addition, the low incidence of the disease has restricted the feasibility of prospective randomized controlled trials, resulting in limited evidence on the effectiveness of systemic adjuvant therapies, which remains largely based on anecdotal reports and isolated clinical experiences.

## Conclusions

Tibial adamantinoma illustrates the contradictions of slow-growing biology with malignant potential. Despite excellent survival rates relative to high-grade sarcomas, the potential for late local recurrence and metastasis requires continuous monitoring throughout the patient's life. Radical en bloc resection with clear margins is essential for oncological management, since insufficient excision is significantly associated with recurrence and systemic dissemination. Reconstruction durability after substantial tibial excision is crucial. Endoprostheses give instant stability but need more revisions than biological techniques like vascularized fibular grafts, which offer better long-term integration and functional durability. Distraction osteogenesis enhances reconstructive options; however, problems at the docking site usually require further fixation and grafting. Functional results across modalities are usually favorable, with most patients attaining independent ambulation and maintaining quality of life; however, reconstructive longevity ultimately determines long-term success, illustrating the need for personalized planning that considers patient age, activity level, defect size, and expected lifespan. This review illustrates the careful balance between oncological surveillance and reconstructive planning in the treatment approach of tibial adamantinoma. Disease control and long-term functional outcomes require a multidisciplinary approach, with lifelong follow-up to accommodate treatment modifications. This strategy enables patients to remain recurrence-free with recovery of durable limb function over the long term.

The clinical management of tibial adamantinoma requires a comprehensive long-term strategy due to its biphasic histological characteristics and the potential for recurrence many years, or even decades, after initial treatment. The findings of this review suggest that a limited disease-free interval should not be interpreted as definitive evidence of cure. Rather, patients require lifelong surveillance within a multidisciplinary framework aimed at the early detection of local recurrence and distant metastatic disease. Furthermore, the review highlights the importance of individualized surgical planning, with an emphasis on achieving durable mechanical reconstruction to preserve function, maintain mobility, and optimize long-term quality of life. This is particularly relevant given the substantial risk of complications associated with limb-salvage procedures.
